# Survey on Addressing the Information and Support Needs of Jewish Women at Increased Risk for or Diagnosed with Breast Cancer: The Sharsheret Experience

**DOI:** 10.3390/healthcare3020324

**Published:** 2015-05-22

**Authors:** Kenneth P. Tercyak, Elana Silber, Andrea C. Johnson, Adina Fleischmann, Sarah E. Murphy, Darren Mays, Suzanne C. O’Neill, Christina M. Sharkey, Rochelle Shoretz

**Affiliations:** 1 Division of Population Sciences, Lombardi Comprehensive Cancer Center, Georgetown University Medical Center, Washington, DC 20007, USA; E-Mails: acj36@georgetown.edu (A.C.J.); sem254@georgetown.edu (S.E.M.); dmm239@georgetown.edu (D.M.); sco4@georgetown.edu (S.C.O.); cms278@georgetown.edu (C.M.S.); 2 Sharsheret, Teaneck, NJ 07666, USA; E-Mails: esilber@sharsheret.org (E.S.); afleischmann@sharsheret.org (A.F.); rshoretz@sharsheret.org (R.S.)

**Keywords:** breast cancer, genetic risk, education, support, quality of life

## Abstract

Approximately 12% of women living in the United States will be diagnosed with breast cancer during their lifetimes. While all women face formidable challenges posed by the threat of living with or at increased risk for breast cancer, those of Ashkenazi Jewish descent face additional challenges owing to higher *BRCA1/2* mutation prevalence in this population. Amidst calls for population-based screening for hereditary breast cancer risk, much can be learned from the experiences of Jewish women about their needs. The present study is a secondary analysis of psychoeducational program satisfaction and evaluation data previously collected by a community organization dedicated to serving women of all Jewish backgrounds facing, or at risk for, breast cancer. Among respondents (*n* = 347), over one-third were referred to the organization by family or friends, most often after a cancer crisis. Of the information and support resources offered, the greatest level of engagement occurred with the one-on-one peer support and health care symposia resources. Respondents endorsed high levels of satisfaction with the programs and services, and a strong desire to give back to the community. These data suggest that culturally-relevant information and support services for Jewish women could be scaled-up for larger dissemination to meet the anticipated needs in this special population.

## 1. Introduction

Approximately 12% of women in the United States (USA) will be diagnosed with breast cancer during their lifetimes [1]. Based on USA population data from 2007–2011, the incidence rate of breast cancer is estimated at 125 per 100,000 women per year with a mortality rate of about 22 per 100,000 women per year [1]. Risk factors for breast cancer include family history of breast or ovarian cancer, mutations in major breast cancer-causing genes (e.g., *BRCA1* and *BRCA2*; *BRCA1/2*), and Central or Eastern European Jewish heritage (primarily Ashkenazi Jewish descent), radiation therapy to the breast or chest at a young age, as well as other breast health conditions or dense breasts [2]. It is further estimated that about 5%–10% of all breast cancer cases are attributable to a *BRCA1*/*2* mutation [3], and women identified with a *BRCA1*/*2* mutation face significantly increased lifetime risks of developing breast cancer [4,5]. Breast cancer is often more aggressive and with poorer outcomes in young women [6].

Although overall breast cancer survival rates are improving, there remain significant health and quality of life impacts among those at increased risk, those who are newly diagnosed and at young ages, and those surviving with the disease [7]. Importantly, women of Ashkenazi Jewish descent confront special challenges adjusting to breast cancer and hereditary breast cancer risk in its context. For instance, *BRCA1/2* mutation prevalence is higher in this population than it is in the general population of women from non-Ashkenazi Jewish backgrounds (1:40 *vs.* 1:400). Regarding psychological outcomes, a study by Metcalfe and colleagues found that among Jewish *BRCA* mutation carriers, self-reported cancer-related distress increased significantly in the year following genetic testing but did not change among women without a mutation [8]. Among Jewish women with breast cancer, positive effects of religion on coping with the disease have been observed [9], and the involvement of Rabbinic and Jewish community leaders may facilitate health care providers’ understanding of religious and cultural issues [10].

In order to potentially act on available risk-reducing and breast cancer management options, it is necessary to first identify women who may be at increased risk for breast cancer owing to family history, *BRCA1/2* mutations, and other factors. Clinical and public health approaches to screening and testing for *BRCA1*/*2* mutations have evolved over the past two decades. Recommendations focus on incorporating family history-taking and conducting a cancer risk assessment for patients, and subsequently deciding on whether to proceed with genetic testing [11]. Yet, genetic testing of all women in the Ashkenazi Jewish population may be viable, regardless of their breast cancer family history, and without adverse psychological and quality of life impacts [12]. The cancer prevention and control field has begun to consider the risks and benefits of regularly screening groups known to be at higher risk of breast cancer, specifically those of Ashkenazi Jewish descent [13,14,15]. Concerns have been expressed that this type of targeted screening of Jewish women may serve to heighten group differences, resulting in group discrimination by exaggerating genetic differences leading to unequal access to testing and treatment [16,17]. However, many Ashkenazi Jewish women have embraced the potential gains to be realized by extending cancer genetic testing to the population, citing increased ethnic disease risk awareness and stronger sense of community [18]. Other research has found that Ashkenazi Jewish women are motivated to undergo genetic testing by their desire to contribute to the health of their family and others in their community [19]. Population-based screening for Ashkenazi Jewish women age 30 years or older has also been shown to be cost-effective [20,21]. With these considerations in mind, the community’s information and support needs are complex and expanding.

As such, it is critical that women facing a risk of hereditary breast cancer be adequately educated and counseled along their journey, both medically and psychosocially, before and after learning about their cancer risk and disease status [22,23,24]. Given the limited time and resources of most cancer care specialists to offer culturally-targeted information to women considering genetic counseling and testing for *BRCA1/2* mutations and those already diagnosed with breast cancer, such resources can be offered outside of the health care setting to augment professional services. As described in widely-accepted conceptual models [25,26,27,28,29], health-related quality of life determinants including symptom status and health perceptions are influenced by psychological and social supports. This notion is grounded in social support constructs, including emotional (expressions of empathy and trust), instrumental (tangible aid and services), informational (suggestions and information), and appraisal (information useful for self-evaluation) supports [30]. Furthermore, as indicated in the PEN-3 cultural model, cultural identity, relationships and expectations, and cultural empowerment are domains in which to anchor supports and interventions seeking greater community engagement [31]. Work addressing cultural identity and socioemotional supports points to comprehensive models to increase program engagements and improve health outcomes for patients with cancer [32,33]. However, a more detailed understanding is necessary for Jewish women living with or at risk for breast cancer to fully ascertain the availability, content, and usefulness of such support services. These insights would allow for improved effectiveness and cultural relevancy for services such as national peer support and patient navigation networks, health education efforts, and programs related to breast cancer treatment and survivorship.

As noted by the Centers for Disease Control and Prevention, evaluation is the systematic application of scientific methods to assess programs, services, and resources organized by community agencies and the like [34]. Given the national and international prominence of breast cancer as a leading women’s health issue, it is essential that breast cancer resources be evaluated both for the general population and special populations, including Ashkenazi Jewish women [35], so that their effectiveness can be optimized and program accomplishments can be more widely disseminated [36].

In this work, we report on the results of an outcome evaluation conducted by Sharsheret, a not-for-profit community-based organization whose mission is to connect Jewish women of all backgrounds with no-cost, culturally-targeted resources at all points along their cancer journey: from risk prevention, to diagnosis and during treatment, to survivorship care. The name Sharsheret is Hebrew for chain. The chain symbolizes connections made among Jewish women, families, and communities facing breast cancer together. Through Sharsheret’s outreach activities, each connection made is a link that strengthens the chain of information and support for constituents nationwide. The evaluation included measures and metrics of community referral practices to Sharsheret, service and program engagement and satisfaction, and additional resource needs among the target group of Jewish women at risk for or living with breast cancer.

## 2. Methodology

### 2.1. Overview

This is a secondary analysis of anonymous annual self-report surveys collected by Sharsheret. The purpose of the annual survey is to ascertain satisfaction and other outcome data about Sharsheret’s psychoeducational programs and services. The survey included a combination of both open- and closed-ended items, with Likert-type responses. Surveys were program-specific and developed by the organization. The study protocol was reviewed and exempted by the Institutional Review Board at Georgetown University Medical Center. This exemption is in accordance with Title 45 of the Code of Federal Regulations Part 46.102 (d), as well as Part 46.102 (f). Briefly, upon contacting Sharsheret, support staff members direct constituents to one or more information and support programs and services (*i.e.*, resources) that are culturally-targeted and based on constituent needs noted during telephone or E-Mail interactions. The present analysis focuses on an evaluation of six of Sharsheret’s signature resources, each of which is described below. These resources are: (1) Peer Support Network; (2) Embrace; (3) Busy Box^®^; (4) Best Face Forward^®^; (5) Genetics for Life^®^; and (6) Health Care Symposia.

#### 2.1.1. Peer Support Network

The Peer Support Network connects women, newly diagnosed with or at high risk of developing breast cancer, with one-on-one trained volunteer peer supporters who share similar diagnoses and experiences. The peer supporter provides tips and resources for coping, emotional support, and navigating the shared experience together.

#### 2.1.2. Embrace

Embrace is designed to meet the needs of women who are living with metastatic breast cancer. The program offers one-on-one support, and primarily includes a trained mental health professional who coordinates and facilitates telephone-based support group calls. The group calls enable constituents to share their experiences together. Embrace constituents are connected with treatment-related materials addressing specific needs and concerns of Jewish women living with advanced cancer.

#### 2.1.3. Busy Box

A Busy Box offers support for constituents facing breast cancer while simultaneously raising young children (under 13 years). Jewish parents of young children face unique concerns, such as communicating with their children about their cancer diagnosis and possible hereditary implications for the family. The program is tailored to the constituent’s expressed needs and concerns, as well as the age and gender of child(ren) and potential religious observances. The program includes pamphlets related to coping with a breast cancer diagnosis and family communication. Age-appropriate toys and games are also provided to children for constituents undergoing treatment.

#### 2.1.4. Best Face Forward

Best Face Forward provides resources and materials addressing the cosmetic side effects of radiation and chemotherapy treatment for Jewish women. Informational materials and a kit are provided, which includes brochures about body image and related topics.

#### 2.1.5. Genetics for Life

Genetics for Life addresses the concerns of Jewish women at higher risk of developing hereditary breast and/or ovarian cancer. This program provides constituents with information related to deleterious mutations in the *BRCA1*/*2* genes, which are known to impact women of Ashkenazi Jewish descent. This information is delivered through various channels: (1) inquiring to the organization’s “Genetic Hotline”; (2) matching with another woman for a one-to-one telephone call; and (3) reviewing an educational booklet, *Your Jewish Genes*, exploring hereditary breast cancer and ovarian cancer risk in the Ashkenazi Jewish community.

#### 2.1.6. Health Care Symposia

Sharsheret’s Health Care Symposia educate Jewish women nationwide on a spectrum of issues within breast cancer prevention and treatment. Through national teleconferences, seminars, and webinars, issues are presented through a culturally-relevant lens. Symposia topics include discussions on physical activity and nutrition, fertility and treatment side effects, spirituality and Jewish holidays/cultural and life-cycle events, as well as interpersonal relationship issues. Symposia topics are generated from within the constituent group through symposia evaluations, focus groups, and staff recommendations based on communications with constituents.

### 2.2. Sampling and Constructs

The target population for this analysis included constituents served by Sharsheret who were at risk for and/or living with breast and/or ovarian cancer, and with contact information maintained in an administrative database. At the end of each calendar year of service (2010, 2012, 2013), constituents who had provided an E-Mail address to Sharsheret were sent an invitation and follow-up invitation reminder through an E-Mail marketing service to complete a program evaluation by clicking on a URL that was not unique to the intended recipient. (That is, all constituents received the same URL.) This E-Mail marketing service does not have the capacity to monitor individual invitation deliveries, but is able to track responses. Based upon information made available to the study team, 36% of E-Mailed invitations distributed to constituents were opened, 28% of opened invitations clicked-through to the survey, and 89% of opened surveys were completed. This resulted in an overall E-Mailed survey response rate of 9%, which is not uncommon among Internet surveys. Sharsheret does not proactively update constituents’ contact information: contact information is updated upon the request of constituents, or at the time of subsequent contact with the organization. Thus, it is difficult to determine the survey’s exact response rate as undeliverable surveys could not be traced or eliminated from the denominator.

The survey itself was administered online through the provided URL that linked to a separate secure survey research platform. Inspection of the survey platform data indicated that 97% of surveys were completed from unique Internet Provider (IP) addresses. Multiple responses from the same IP address might have indicated that a single constituent responded to the survey twice, or that multiple constituents accessed the survey from within the same computer network (e.g., home, worksite, public library, community organization building), which could not be further determined. In only one instance did we identify that the same IP address was used to complete an annual survey for two consecutive years. This suggested that the vast majority of surveys likely reflected the experiences of unique survey respondents. For those constituents who did not provide an E-Mail address but who provided a physical mailing address, a paper survey was sent by regular mail. (No data were available for 2011 as Sharsheret was engaged in a nationwide needs assessment survey for young breast cancer survivors.)

Survey domains included methods of referral to Sharsheret, engagement and satisfaction with Sharsheret resources, and inquiries about information and support needs among Jewish women. Demographic characteristics of survey respondents (e.g., age, marital status, religious affiliation), as well as clinical characteristics (e.g., cancer status, age at diagnosis) and family composition (e.g., number and ages of children) were assessed for the 2013 annual survey only. These data are provided on this subset of respondents as they are likely representative of survey respondents overall.

### 2.3. Analyses

Descriptive statistics were generated to characterize the sociodemographic and clinical characteristics of the 2013 subsample (Table 1), and to define referral to and level of constituent engagement with each of the six resources by calendar year (Table 2). Where appropriate, program-specific satisfaction items were adapted to derive composite summary scores. Each of Sharsheret’s programs and services were evaluated by inquiring about participant agreement with a series of 4 to 10 affirmative satisfaction statements for each resource. Due to differences in survey Likert-type response option formats across the three years (e.g., Agree, Disagree *vs.* Agree, Somewhat agree, Somewhat disagree, Disagree) some variables were dichotomized to reflect constituents’ survey responses for data harmonization purposes. Only those survey items that were consistently administered across all three years of survey data collection were included in the analysis. Overall satisfaction with services provided is represented by the mean of all individual program satisfaction scores for each participant, excluding programs that the participant did not engage in. These scores are stratified by each resource and provided overall (Table 3). Respondents who indicated that survey items were “not applicable” to them, most likely owing to the fact that they did not engage with that resource, were excluded from the analysis of resource satisfaction. Lastly, the analysis included reviewing the proportion of survey respondents who suggested that additional resources be provided by Sharsheret in the future.

**Table 1:** 2013 Survey Respondent Characteristics (*n* = 133).

	Mean	SD	n	%
**Demographics**				
Age	49.03	10.26		
Marital Status				
Married/Living as Married			93	69.92
Single			23	17.29
Divorced/Separated/Widowed			14	10.53
No Response			3	2.26
Religious Affiliation				
Jewish			117	87.97
Non-Jewish			12	9.02
No Response			4	3.01
**Clinical Characteristics**				
Age at Cancer Diagnosis (if applicable; years)	44.77	9.47		
Cancer Status at Survey *				
Breast or Ovarian Cancer Survivor			87	65.41
At Risk or *BRCA1/2* Mutation Carrier			33	24.81
Living with Breast and/or Ovarian Cancer			19	14.29
Recently Diagnosed with Breast Cancer			15	11.28
No Response or Other			14	10.53
**Family Composition**				
Number of Children				
0			33	24.81
1 or more			95	71.43
No Response			5	3.76
Age(s) of Child(ren) * (if applicable; years)				
0–17			64	48.12
18 or Older			58	43.61
**Role at Sharsheret**				
Peer Supporters			23	17.29

* Observations do not total to 100% due to multiple responses.

**Table 2:** Engagement with Evaluated Resources, Stratified by Survey Year.

	Resources/Year							Resources/Constituent
**Year (*n*, %)**	Peer Support Network	Embrace	Busy Box	Best Face Forward	Genetics for Life	Health Care Symposia	Other	Total
	*n* (%)	*n* (%)	*n* (%)	*n* (%)	*n* (%)	*n* (%)	*n* (%)	*M* (*SD*)
2010 (101, 29)	64 (31)	4 (2)	23 (11)	16 (8)	18 (9)	47 (22)	37 (18)	2.06 (1.28)
2012 (113, 33)	58 (26)	9 (4)	29 (13)	23 (10)	27 (12)	47 (21)	30 (13)	1.97 (1.31)
2013 (133, 38)	58 (21)	7 (3)	28 (10)	30 (11)	25 (9)	37 (13)	96 (34)	2.11 (1.43)
Total (347, 100)	180 (25)	20 (2.81)	80 (11)	69 (10)	70 (10)	131 (18)	163 (23)	2.05 (1.35)

**Table 3:** Satisfaction with Evaluated Resources.

Resource (# of Satisfaction Items)	Satisfaction Score *M* *(SD*)
Peer Support Network (3 items)	95.2 (0.17)
Embrace (5 items)	100.0 (0.00)
Busy Box (3 items)	95.5 (0.19)
Best Face Forward (4 items)	92.9 (0.26)
Genetics for Life (4 items)	97.9 (0.71)
Health Care Symposia (2 items)	98.0 (0.12)
Overall Satisfaction (per constituent)	96.3 (0.13)

## 3. Results and Discussion

### 3.1. Sample Characteristics

The study sample included 347 survey respondents. Again, only those who completed the 2013 survey were asked to report on sociodemographic and individual-level information (*n* = 133; 38% of total). As shown in Table 1, the mean age of respondents was 49 years old. These individuals were mostly married or living as married, and the vast majority identified their religious affiliation as Jewish. Most respondents had one or more children. As a group, they mostly identified as breast cancer survivors, about one-quarter were *BRCA1/2* mutation carriers, and about one-quarter of respondents were also trained as a Sharsheret peer supporter.

#### 3.1.1. How Are Constituents Referred?

More than one-third of constituents initially contacted Sharsheret through a recommendation from a family member or friend (34%), and most commonly after a new breast cancer diagnosis (44%). Otherwise, constituents located the organization through a health care provider, online, or through another organization or outreach event.

#### 3.1.2. What Programs Do Constituents Engage With?

Sharsheret offers multiple resources to Jewish women of all backgrounds. Annual survey data indicate that constituents engaged with an average of two resources assessed, as shown in Table 2. As engagement with multiple resources was common, not all percentages sum to 100%. Within the six resources offered by the organization across all years surveyed, the highest levels of engagement were reached within two resources: (1) Peer Support Network, including one-on-one calls with tips for coping and a shared experience; and (2) Health Care Symposia, including informational seminars, teleconferences, and educational webinars about breast cancer prevention and treatment. Together, these two resources constituted >40% of the evaluation data collected across the three years surveyed. Reported engagement in Busy Box, Best Face Forward, and Genetics for Life all yielded similar numbers and comprised approximately 30% of the evaluation data. The number reporting engagement in Embrace (for women with advanced or metastatic cancer) was low, accounting for <3% of the evaluation data overall. New resources were offered by Sharsheret yearly, and these resources were included in the “Other” category of Table 2. Examples include Financial Wellness and Thriving Again survivorship programs (2013). Constituents who engaged with Sharsheret’s Peer Support Network in the role of peer supporters were also included in the “Other” category as these women initially received the resource. A negligible number of constituents did not report engaging with any of the resources. This was either due to missing data or engaging with Sharsheret in some capacity outside the scope of the evaluated resources. Overall, the “Other” category indicates Sharsheret’s progress toward expanding their resource offerings over time.

#### 3.1.3. How Satisfied Are Constituents with the Resource(s) they Engaged With?

Composite satisfaction scores were derived from each evaluated resource on the corresponding annual survey. The data revealed that the majority of survey respondents reported very high levels of satisfaction with the resources from Sharsheret that they engaged with (scale = 0–100, lowest to highest satisfaction; Table 3).

It is important to note that all evaluated programs and services scored at 92% satisfaction or higher: Sharsheret’s resources received an average rating of 96% satisfaction. Respondents for the Embrace, Health Care Symposia, and Genetics for Life resources tended to endorse the highest levels of satisfaction, but these satisfaction scores were not statistically different from the others.

#### 3.1.4. What Additional Needs Arise for Constituents?

**Figure 1:**
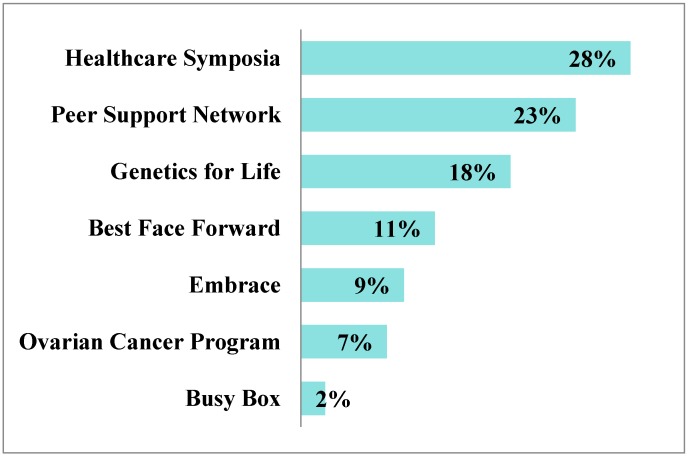
Future Information and Support Needs.

Respondents were also provided with the opportunity to request additional information related to future Sharsheret programs and services. Data indicate that additional information and support were most commonly requested for the Health Care Symposia and Peer Support Network, followed by Genetics for Life, Best Face Forward, and Embrace (Figure 1). Similar to resource engagement, constituents were able to inquire about multiple resources.

When respondents were asked if they would you like to become a member of Sharsheret’s Peer Support Network in the future (who volunteers and trains to be paired with a caller for the Peer Support Network), over one-half (53%) of respondents expressed interest in doing, which is further testament to the success of the resource and the interests of Jewish women to give back to their communities.

## 4. Conclusions

The purpose of this paper was to describe how Jewish women facing the risk of or surviving with breast cancer seek-out, utilize, and evaluate information and support provided by a not-for-profit community-based organization (Sharsheret) dedicated to culturally-targeted, no-cost resources. Annual self-reported survey results indicate that constituents initially connected with Sharsheret after referral from a friend or family member while they were in a time of medical crisis or transition, such as a new breast cancer diagnosis. Typically engaging with two or more resources, constituents were overwhelmingly satisfied with the information and support programs and services they encountered. The two resources with the highest levels of engagement were Sharsheret’s one-on-one peer support network and its health care symposium format designed to educate Jewish women about breast cancer prevention and treatment. Thus, as an organization, Sharsheret is meeting this special population’s needs for resources that deeply understand and attend to cultural and religious beliefs and values held by Jewish women of all backgrounds. Each of the resources was evaluated as highly satisfying among survey respondents. These resources share a common focus on meeting the information and support needs of Jewish women about breast cancer, but differ in their communication modality and format. By offering a range of programs and services, Sharsheret is better able to target and tailor their programs to constituents across the cancer prevention-control spectrum.

Support, including social support, was provided through the Peer Support and Embrace programs, and is commonly demonstrated in individual and group-based programming [37]. Information was provided through the Health Care Symposia, print, and other materials. These align well with interventions seeking to empower individuals to be informed consumers and stakeholders in their own health care [38]. These types of resources augment larger conceptual models showing the interrelationships among social networks, social support, and health outcomes [30]. In particular, factors that impact health outcomes include: individual coping resources (e.g., problem-solving abilities, access to individuals and information, perceived control) and organizational and community resources (e.g., community advocacy and capacity). These components interact with available social supports and individual stressors to impact physical, mental, and social health. A review of these interconnected relationships lends itself well to shaping resources for high risk and breast cancer affected populations. Many constituents requested to become peer supporters themselves and maintain a connection with Sharsheret over time. Women in this sample also desired an opportunity to give back to the community that assisted them. In effect, Sharsheret is building the foundation for an intrinsically motivated, community-based, social support network—and realizing its mission of linking together Jewish women, families, and communities impacted by breast cancer. This effort has the potential to greatly improve constituents’ health outcomes. Further resources to enable additional peer supporter training seem warranted in order to scale-up Sharsheret’s programs and services to the broader community at-large.

The findings from this evaluation should be considered in light of several important limitations. First, evaluation data were generated from a relatively small, non-representative convenience sample, with the potential influence of multiple selection biases towards those who were more satisfied/engaged with Sharsheret’s resources. Importantly, just over one-quarter of the surveyed sample self-identified as being peer supporters of the organization—meaning they had received Sharsheret’s services themselves, and then went on to help other constituents. Second, the total number of surveys distributed electronically and to valid recipient addresses could not be accurately determined due to tracking restrictions, which adversely impacted our ability to derive the survey’s response rate. Regarding completed surveys, 97% were linked to unique IP addresses, suggesting they were completed by independent respondents, but this cannot be entirely verified. Third, with some minor differences in Likert response structure by year, only those items consistently present in the annual outcome surveys were included. Consequently, while the review contains data merged from all three years, demographic data were only available for one evaluation year. Also, survey items were agency-specific and did not include well-validated scales and measures. Finally, we could not stratify our analysis based on self-reported clinical characteristics, such as cancer status at the time of the survey, because such data were unavailable for the entire sample. Further examination of this population and additional validation of results over time is necessary.

The high level of satisfaction paired with requests for additional information and support among this population present an opportunity to deepen and expand such resources to fill gap areas in the care of Jewish women affected by or at high risk of developing breast cancer. These needs can be met by organizations dedicated to increased breast cancer awareness, empowerment, and service. Community health workers and patient navigators are increasingly becoming integrated into cancer care and preventive medicine. Community engagements that focus on genetic and environmental determinants of cancer can favorably impact upon utilization of screening and genetic testing for familial breast cancer risk. These services should be evidence-based, and grounded in interpersonal and ecological theories to consider constituents’ self-efficacy and structural facilitators and barriers of health promotion and health behavior. Early adopters within communities may facilitate increased adoption among others later on. Embracing cultural targeting allows for support services to be highly salient to the intended community of interest, such as Jewish women facing the prospect of or living with breast cancer. This is particularly timely if and when population-based screening for breast cancer genes becomes commonplace and more Jewish women are seeking information about hereditary breast cancer or otherwise affected by breast cancer in their lives.
